# MicroRNAs作为肺癌的治疗靶标

**DOI:** 10.3779/j.issn.1009-3419.2010.12.17

**Published:** 2010-12-20

**Authors:** William CS CHO, 娟 南

**Affiliations:** 1 Department of Clinical Oncology, Queen Elizabeth Hospital, Hong Kong; 2 天津医科大学总医院，天津市肺癌研究所，天津市肺癌转移与肿瘤微环境重点实验室; 3 香港特别行政区 伊利沙伯医院 临床肿瘤科

**Keywords:** miRNA抑制剂, 锁核酸, 肺癌, miRNA（microRNA）, miRNA疗法, 治疗靶标

## Abstract

肺癌是当今世界的主要致死原因之一，亟待新的治疗方法。近年来，microRNAs已成为调节基因表达的关键因子之一。许多研究表明，microRNAs几乎参与肺癌癌变过程的每一阶段，包括肿瘤的发展、细胞凋亡、癌细胞的侵袭和转移，以及抗癌药物的耐药。MicroRNA的强制表达或抑制可调节癌变过程中的生物学改变，表明了microRNAs在肺癌中具有治疗潜能。本社论总结调节肺癌癌变过程的一些重要microRNAs的最新报道，并阐释其作用机制，介绍一些调控microRNAs作用的方法，并探讨了microRNAs作为肺癌治疗靶标的前景。

## 1 前言

近年来，microRNAs（miRNAs）已成为基因表达的强力调控因子^[1]^。为了开发新型miRNA疗法，鉴定miRNA靶标为当前的首要目标。本文就miRNAs作为肺癌治疗靶标的几个方面作一探讨。

## 2 MiRNAs与肿瘤的发展

许多研究表明，miRNAs几乎参与肺癌癌变进程的每一阶段，包括肿瘤的发展^[2]^。Cho等^[3]^发现，在*EGFR*突变的肺癌细胞中，肿瘤抑制因子*miR-145*的功能恢复可阻遏癌细胞的生长，*miR-145*有可能成为肺腺癌的治疗靶标。Liu等^[4]^报告，在肺癌中，*miR-31*是一个致癌miRNA（oncogenic miRNA, oncomir），敲除*miR-31*可通过增加肿瘤抑制基因*LATS2*和*PPP2R2A*的表达以剂量依赖性方式显著抑制癌细胞的生长和致瘤性。Ebi等^[5]^发现，在*RB*失活的肺癌细胞中，*miR-17-92*的过表达可能在对抗DNA损伤的产生中发挥微调作用，因此，与损伤DNA的化疗药物联合，*miR-17-92*可能成为消除过量DNA损伤的治疗靶标。

## 3 MiRNAs与细胞凋亡

越来越多的研究清楚表明，miRNAs具有在不同水平调节细胞凋亡的潜能和关键作用。Duan等^[6]^发现，*miR-34a*是PRIMA-1诱导肺癌细胞凋亡网络中的重要组分之一。在携带突变型*p53*的癌细胞中，敲除*miR-34a*可降低PRIMA-1诱导的细胞凋亡率。Incoronato等^[7]^发现，*miR-212*是一个肿瘤抑制因子，可通过负性调节抗凋亡蛋白PED/PEA-15的表达而增加非小细胞肺癌（non small cell lung cancer, NSCLC）肿瘤坏死因子相关凋亡诱导配体（tumor necrosis factor-related apoptosis inducing ligand, TRAIL）治疗的敏感性。

## 4 MiRNA治疗

强制miRNA的表达或抑制可调节癌变中的生物学改变，这突显了miRNAs在肺癌中的治疗潜能^[8]^。Trang等^[9]^证明在NSCLC小鼠模型中，外源性注入肿瘤抑制因子*let-7*形成的肿瘤可明显减轻在体肿瘤负荷。他们的结果表明，miRNA替代疗法是一种有前景的肺癌治疗方法。Wiggins等^[10]^利用化学合成的肿瘤抑制因子*miR-34a*和脂质输送载体开发了一种可阻断NSCLC小鼠模型的肿瘤生长的治疗方案。无论局部或全身用药，该治疗方案均有效，耐受性好，且不会诱发免疫应答。Chen等^[11]^也开发了一种肿瘤靶向性单链抗体片段修饰的脂质体-多聚阳离子-透明质酸纳米颗粒载体，可将*miR-34a*经体循环运输至实验性肺转移鼠黑色素瘤细胞内。经由纳米微粒运输的*miR-34a*可显著下调转移性肿瘤中survivin的表达，并可减轻肺脏的肿瘤负荷。

## 5 侵袭和转移性肺癌中的miRNA治疗靶标

癌症最致命的特点是其具有侵袭和转移的能力。Garofalo等^[12]^发现，在侵袭性NSCLC细胞中，过表达的*miR-221*和*miR-222*通过靶向作用于肿瘤抑制因子*PTEN*和*TIMP3*可诱导TRAIL耐药，并通过激活AKT通路和金属肽酶来促进细胞转移。他们进一步发现，癌基因MET通过转录因子*c-Jun*参与*miR-221*和*miR-222*的活化。Muniyappa等^[13]^发现*miR-29a*对离体肺癌细胞具有显著的抗侵袭和抗增殖作用。*MiR-29a*具有抗癌miRNA的作用，这一作用可能通过多个蛋白（包括RAN，*RAS*癌基因家族的一员）在分子水平的转录后微调来介导。Gibbons等^[14]^表明，*miR-200*的强制表达可消除转移性肺腺癌细胞的上皮间质转化、侵袭和转移的能力。*MiR-200*的表达可调节肿瘤细胞的转移，其表达随其通路前后因子的细胞外信号而改变。Ma等^[15]^发现，在荷高转移性细胞的小鼠中采用miRNA抑制剂沉默*miR-10b*可显著提高*Hoxd10*的水平，并明显抑制肺转移的形成。正常动物可耐受*miR-10b*的miRNA抑制剂，*miR-10b*有可能成为新型抗转移药物开发的潜藏标靶。

## 6 MiRNA在抗癌药物耐药中的作用

越来越多证据表明，某些miRNAs可靶向作用于与药物敏感性相关的基因，从而导致癌细胞对抗癌药物的敏感性的改变^[16]^。Guo等^[17]^发现，将*miR-134*的类似物转染至耐药小细胞肺癌（small cell lung cancer, SCLC）细胞中可明显提高其对抗癌药物顺铂、依托泊甙和阿霉素的敏感性。在耐药SCLC细胞中，*miR-134*通过诱导G_1_期阻滞可提高细胞生存率，并可下调MRP1/ABCC1蛋白的表达。Zhu等^[18]^表明，*miR-181b*的过表达可降低BCL2蛋白水平，并可提高多药耐药肺癌细胞对顺铂诱导细胞凋亡的敏感性。Galluzzi等^[19]^也发现，在NSCLC细胞中，pre-*miR*- *181a*和pre-*miR-630*可分别增强或减弱顺铂诱发的细胞凋亡。它们可同步调节细胞凋亡内在通路的线粒体和线粒体后阶段，包括Bax低聚反应、线粒体跨膜电位的消失以及caspase-9和caspase-3蛋白分解作用的完成。MiRNA调控网络亦可能是克服肺癌辐射耐受的潜在治疗靶标。Oh等^[20]^发现，*let-7a*的过表达可降低K-Ras的表达，并可提高携带活化K-Ras信号的肺癌细胞的辐射敏感性。Lin28为*let-7*的抑制剂，Lin28的抑制可降低K-Ras的表达，并可提高携带*K-RAS*突变的肺癌细胞的辐射敏感性。

## 7 专家观点

显然，单一miRNA的治疗性抑制剂或类似物可同时靶向作用于相似通路和信号级联中的多个基因。目前miRNAs作为新型疗法的潜能正处于研究中。MiRNA疗法的主要难题是稳定性、安全性以及如何有效运输至组织或器官内的特定细胞。近年来，先进技术使癌症的分子、细胞、临床以及治疗研究取得了重大成果^[21]^。目前有多种在研的通过调控上调miRNA表达的方法，如miRNA抑制剂、小分子和miRNA海绵。MiRNA抑制剂是被广泛采用的调节在体miRNA水平的方法，包括2’-O-甲基反义寡核苷酸和锁核酸（locked nucleic acid, LNA）反义寡核苷酸。有研究小组发现，小分子亦可用于调节特定miRNA的功能^[22]^。MiRNA海绵则是另一项利用表达miRNA靶向位点的载体清除miRNA并阻止其调节天然靶点的技术^[23]^。此外，还有一些方法可以模拟或重新表达下调的miRNAs，如脂质型类似物和腺相关病毒载体。

然而，miRNA疗法要想成为癌症治疗切实可行的选择，尚需克服许多难题。MiRNA虽有可喜的治疗潜能，其随之而来的脱靶效应却不容忽视。整合载体能增补长寿基因，但可引起临近整合部位的癌基因激活所致的潜在致癌性风险的增加^[24]^。移除慢病毒载体中的长末端重复序列增强子元件并添加内部启动子可解决这一难题^[25]^。尽管这些治疗方法的开发尚未成熟，但跨学科的纳米生物技术使得miRNA疗法的相关研究飞速进展^[26]^。

多个机构已经开展了临床前或临床试验，旨在各种癌症（包括肺癌、前列腺癌、肝癌、食道癌、白血病、皮肤癌和肾细胞癌）中应用miRNA疗法^[27, 28]^。最近，已有研究报道了LNA抗miRNA技术在非人灵长类动物中的治疗适应症。采用抑制*miR-122*的LNA修饰的寡核苷酸治疗慢性感染的黑猩猩可持久抑制丙肝病毒血症，且在受试动物中未见病毒耐药反应或副作用^[29]^。这一令人鼓舞的结果强调可将miRNAs从实验室研究扩展至临床转化研究^[30]^。尽管miRNAs作为新型疗法的研发仍困难重重，但已有的发现显示了miRNAs在肺癌治疗中的巨大潜能。

## Declaration of interest

The author states no conflict of interest and has received no payment in preparation of this manuscript.
